# All-in-One Deposition to Synergistically Manipulate Perovskite Growth for High-Performance Solar Cell

**DOI:** 10.34133/2020/2763409

**Published:** 2020-10-14

**Authors:** Yifan Lv, Hui Zhang, Jinpei Wang, Libao Chen, Lifang Bian, Zhongfu An, Zongyao Qian, Guoqi Ren, Jie Wu, Frank Nüesch, Wei Huang

**Affiliations:** ^1^Key Laboratory of Flexible Electronics (KLOFE) and Institute of Advanced Materials (IAM), Jiangsu National Synergetic Innovation Center for Advanced Materials (SICAM), Nanjing Tech University (NanjingTech), 5 Xinmofan Road, Nanjing 210009, China; ^2^Empa, Swiss Federal Institute for Materials Science and Technology, Laboratory for Functional Polymers, Dübendorf CH-8600, Switzerland; ^3^Institut des Matériaux, Ecole Polytechnique Fédérale de Lausanne, EPFL, Station 12, Lausanne CH-1015, Switzerland; ^4^Frontiers Science Center for Flexible Electronics, Xi'an Institute of Flexible Electronics (IFE) and Xi'an Institute of Biomedical Materials & Engineering, Northwestern Polytechnical University, 127 West Youyi Road, Xi'an 710072, China

## Abstract

Nonradiative recombination losses originating from crystallographic distortions and issues occurring upon interface formation are detrimental for the photovoltaic performance of perovskite solar cells. Herein, we incorporated a series of carbamide molecules (urea, biuret, or triuret) consisting of both Lewis base (–NH_2_) and Lewis acid (–C=O) groups into the perovskite precursor to simultaneously eliminate the bulk and interface defects. Depending on the different coordination ability with perovskite component, the incorporated molecules can either modify crystallization dynamics allowing for large crystal growth at low temperature (60°C), associate with antisite or undercoordinated ions for defect passivation, or accumulate at the surface as an energy cascade layer to enhance charge transfer, respectively. Synergistic benefits of the above functions can be obtained by rationally optimizing additive combinations in an all-in-one deposition method. As a result, a champion efficiency of 21.6% with prolonged operational stability was achieved in an inverted MAPbI_3_ perovskite solar cell by combining biuret and triuret additives. The simplified all-in-one fabrication procedure, adaptable to different types of perovskites in terms of pure MAPbI_3_, mixed perovskite, and all-inorganic perovskite, provides a cost-efficient and reproducible way to obtain high-performance inverted perovskite solar cells.

## 1. Introduction

Organometal halide perovskites have recently obtained widespread research interests because of their promising photophysical properties and low-cost manufacturing process, and many optoelectronic applications have been explored in terms of photovoltaics [1], light-emitting diodes [2], lasers [3], photodetectors [4], etc. Among them, photovoltaic devices employing perovskites as light-harvesting materials exhibit the highest commercialization potential due to the astonishingly swift progress in power conversion efficiency (PCE) that has been achieved at both laboratory and industrial levels [5]. Recently, inverted (p-i-n) perovskite devices have attracted tremendous attention due to their ease of manufacturing process, compatibility with stretchable substrates, and suitability for multijunction devices [6]. However, in solution-processed inverted perovskite solar cells, defect states deriving from crystallographic distortion during perovskite crystallization or issues occurring upon interface formation with adjacent layers are of great concern [6–10]. It may be argued that the ionic character of metal halide perovskite semiconductors results in a relatively high tolerance level regarding the density of defects and their impact on PCE [11]. Nevertheless, imperfect interfaces have detrimental impacts on charge diffusion length, nonradiative recombination, hysteresis phenomenon, and operational stability [7, 8, 12–14]. Therefore, it is important to suppress defect states in perovskite solar cells in order to approach the radiative limit of efficiency and to guarantee the lifetime required under operational conditions.

Additive engineering has proven an efficient way to guarantee high-quality perovskite crystal formation [15, 16]. The common approach consists of introducing a small amount of chemicals consisting of some functional elements or special structures into the perovskite precursor. Depending on their chemical structure, the incorporated additives act in different ways. Some functional groups (O–, N–, S– etc.) [16–19] present in the additives can associate with the perovskite precursor (PbI_2_, methylammonium iodide or MAI, etc.) and form some intermediate states to modify the crystallization dynamics and facilitate crystal growth. A second class of additives comprising –C=C– and P=O functional groups can act as cross-linker [20, 21] between adjacent grains to improve the crystal quality and stability. A third type of additives consists of elements that can be incorporated into the crystal lattice, tailoring its tolerance factor and stability [12, 14, 22]. Depending on their effectiveness, additives can be removed after film formation to avoid impurities or remain within the film to coordinate with defect states in the crystal lattice [22]. Additives also act as an interfacial modifier to reduce the energy barrier between the perovskite semiconductor and the contact [23]. However, most of the additives are supposed to play a single role in the above functions or need to be combined step-by-step to achieve different targets that complicate the fabrication process. A rational design of the additive molecular structure advantageously should bring synergistic benefits improving crystal quality as well as eliminating interface electronic defects in an all-in-one deposition method.

Herein, different carbamide derivatives comprising both –C=O and –NH_2_ groups were employed as additives in the preparation of the MAPbI_3_ perovskite films to scrutinize their mode of action related to crystal growth and device performance. By careful choice of the chemical structure and engineering of additive combination, perovskites with enhanced photoluminescence quantum efficiency and interfacial charge transport can be obtained. As a result, a champion efficiency above 21% with prolonged operational stability was achieved for MAPbI_3_ solar cells in an inverted device configuration.

## 2. Results and Discussion

### 2.1. Interaction between Additives and Perovskite Precursors

From the classical LaMer model [24], it is known that one of the key factors for high-quality crystal growth during solution deposition is to trigger a high nucleation rate with optimum nucleus density before crystal growth. This can be achieved by generating intermediate adducts with a suitable molecular weight in the precursor solution. It is generally agreed that stirring the precursor solution for several hours before film deposition is necessary to obtain high perovskite crystal quality via a one-step preparation method [23]. Polar solvents such as dimethylformamide (DMF) and dimethyl sulfoxide (DMSO) can coordinate with PbI_2_ and form intermediate complexes of MAI–PbI_2_–solvent to seed nucleation, while the solvent escape is retarded due to the association between the perovskite precursor and high boiling point solvent, necessitating posttreatments such as of antisolvent dripping [25], solvent annealing [26], vacuum flash evaporation [27], or high-temperature casting [28] (above 90°C). Ideally, such treatments accelerate crystal growth and large crystalline perovskite grain sizes with minimized boundaries are obtained. However, the whole process is complex and time-consuming resulting in a low reproducibility of crystal quality and device performance.

**Figure 1:**
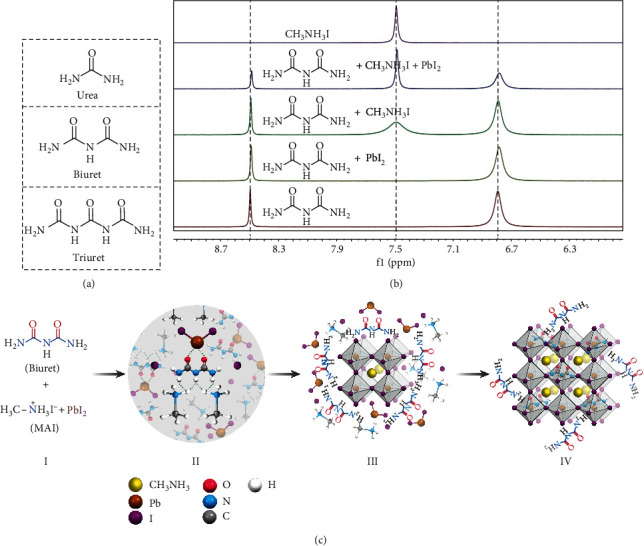
Interaction between the additives and the perovskite precursors. (a) Chemical structures of the additives urea, biuret, and triuret. (b) ^1^H NMR spectra of MAI, MAI+PbI_2_, MAI+PbI_2_+biuret, MAI+biuret, PbI_2_+biuret, and biuret. (c) Schematic illustration of the MAPbI_3_ perovskite crystal growth with the assistance of biuret additive, (I) mixing biuret into the perovskite precursor, (II) association between the precursor and additives and the formation of PbI_2_-biuret-MAI complex, (III) nucleation with additives attached on the nucleus, attracting free monomers (MAI, PbI_2_) for fast crystal growth, and (IV) large perovskite formation with residual additives at the grain boundaries.

Additives of Lewis acids or bases, which are expected to strengthen the interaction with the perovskite precursor by acting as electron acceptors or donors [29, 30], can generate adducts of precursor-additive instead of precursor–solvent complexes. For example, formic acid was found to accelerate perovskite crystallization [31] while halic acids affect the concentration of colloids in the precursor solution [32]. Lewis bases were found to retain the MA cations during solution aging [33], and amino acids were reported to improve device performance and stability [34]. Urea has also been found to form precursor complexes in solution, which lead to large perovskite crystal grains [35, 36]. Therefore, it is of great importance to understand how the incorporated molecules affect perovskite crystal quality. Herein, carbamide derivatives in terms of urea, biuret, and triuret (Figure 1(a)) are selected as additives, and the role of –C=O and –NH_2_ groups present in the additives is scrutinized.

Liquid state ^1^H nuclear magnetic resonance (^1^H NMR) spectroscopy characterizations were performed to investigate how the additives interact with the perovskite precursor in solution. As shown in Fig. S2, when adding urea to the PbI_2_ solution, the typical hydrogen bond signal of the urea was kept constant indicating that the interaction between urea and PbI_2_ is rather weak. In contrast, the characteristic peaks of the H bond of biuret (Figure 1(b)) and triuret showed a slight shift when mixed with PbI_2_ due to the complex formation between the –O=C–NH–C=O– group and PbI_2_ as illustrated in Figure 1(c), II. When mixing the additives into MAI solution, the characteristic peak of the MAI was broadened compared to pristine MAI, suggesting a strong interaction between the additives and MAI. The broadened peak of MAI might be arising from the hydrogen bonding between MAI and –NH_2_, –HN– groups in the additives as indicated in Figure 1(c), II, allowing for the MAI-additive complex formation. It was noticed that the characteristic peak of MAI was sharpened again (to a lesser extent in case of triuret) and a clear peak shift of the H bond in the additives was observed by mixing the additives together with MAI and PbI_2_ (Fig. (S2, S3)), suggesting a strong interaction between the additive and the perovskite precursor. Note that the presence of MAI can enhance the interaction between the additives and PbI_2_. Additionally, it was found that the coexistence of the intermediate –NH– group and the end –NH_2_ group is able to create strong coordination between MAI and the additive (supplementary S1), while the presence of neighboring double –O=C– groups is the key to generate a strong interaction between additive and PbI_2_ by the formation of strongly bonded O–Pb–O chelate structure (Figure 1(c), II). Therefore, the coordination ability between the additives and the perovskite precursor was ranged in the order of urea<biuret<triuret, leading to the possible formation of adducts of MAI···urea···PbI_2_, MAI···biuret–PbI_2_, and MAI–triuret–PbI_2_, respectively, where ··· indicates weak interaction and – indicates strong interaction. The speculated structure of the as-formed MAI···biuret–PbI_2_ adducts is described in Figure 1(c), II.

### 2.2. Additives Assisted Perovskite Crystallization

**Figure 2:**
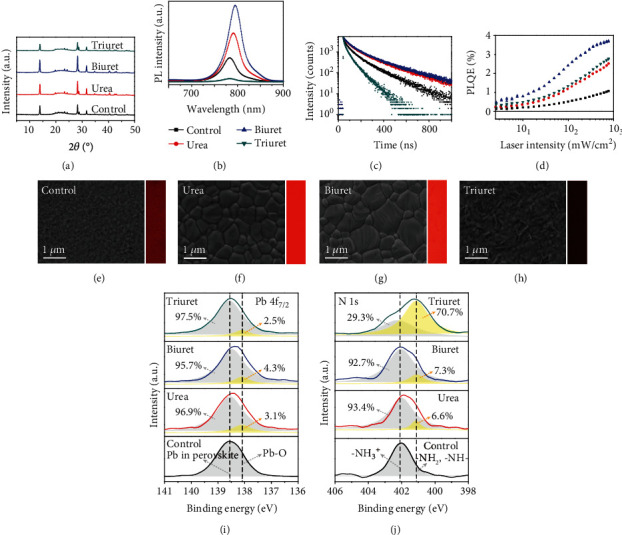
Characterization of perovskite films fabricated with different additives. (a) X-ray diffraction (XRD) spectra. (b) Photoluminescence spectra. (c) Time-resolved photoluminescence spectra. (d) Photoluminescence quantum efficiency. (e–h) Scanning electron microscope (SEM) images followed with photoluminescence microphotographs. (i) Core energy level spectrum of Pb *4f_7/2_*. (j) Core energy level spectrum of N *1*s of perovskite films doped with different carbamide molecules.

To get insight into the effect of the additives on perovskite crystallization, a series of MAPbI_3_ perovskite films were fabricated via a one-step deposition method, where a small amount of additives such as urea, biuret, and triuret was added into the precursor solution. As reported previously, annealing at 100°C for ~10 min in a solvent vapor atmosphere (high-temperature solvent annealing or HTSA) enabled better crystal quality in the preparation of pristine MAPbI_3_ perovskite films [37]. Some obvious differences were observed when the additives were incorporated, that was, the color change from brown to black of the as-deposited perovskite film quickly took place within 5 minutes under a low-temperature (60°C) thermal annealing condition (denoted as LTTA) following the order of triuret~biuret>urea>control as clearly demonstrated in Fig. S4. As confirmed by the absence of the PbI_2_ signal (2*θ* = 12.7°) in the XRD spectrum (Figure 2(a), Fig. S5), all samples treated under both HTSA and LTTA condition were completely transformed into perovskite. No additional diffraction peaks other than those from pristine 3D MAPbI_3_ indicated that there are no residual solvents and lower dimensional crystals in the additive-treated perovskite films [38]. This implies a rapid transformation of the precursor to the perovskite with the assistance of additives, which corroborates previous reports using urea as an additive [35, 36].

As shown in Figures 2(e)–2(h), the pristine MAPbI_3_ film prepared at LTTA exhibited small grains with numerous pinholes (Figure 2(e)). The small grains can grow into larger ones reducing the pinholes in the film when applying HTTA treatment (Fig. S6), indicating that a large energy is required to overcome the barrier for further crystal growth and coarsening. In contrast, when the additives of urea and biuret were added into the precursor solution, the perovskite films demonstrated a large grain size (>1 *μ*m) without pinholes. Almost no obvious morphology difference was observed when using HTSA and LTTA (Figures 2(f) and 2(g), Fig. S6), suggesting a lower energy barrier for perovskite formation and crystal growth. More precisely, the constitutive grains of the perovskite films using biuret as an additive were larger than in the case of urea (Fig. S6), which was attributed to its strengthened coordination ability with the perovskite precursor. However, a significant morphology difference was observed with the addition of triuret. Some small grains and dendritic structures were present on the film surface when prepared using LTTA (Figure 2(h)), and similar to pristine MAPbI_3_, the small grains grew into larger ones when applying HTSA (Fig. S6, S7).

Applying the classical LaMer model on nucleation to solution-processed polycrystalline perovskites, the critical Gibbs free energy (*Δ*G) of the precursor solution spun on the substrates determines the energy barrier for the speed for nucleation and the size of the nucleus [39]. The generation of intermediate MAI-additive-PbI_2_ complexes allowed for the formation of large clusters in solution that can effectively reduce the energy barrier (*Δ*G) and regulate rapid nucleation and crystal growth at the lower annealing temperature. Moreover, the growth of small grains into larger ones suffers from Ostwald ripening [40, 41], which is limited either by the mass transport (diffusion-controlled) or by their attachment on the larger grains (interface-reaction controlled). In the case of using urea or biuret as additives, the strong interaction between the additives and the precursor drives the free “monomers” (PbI_2_ and MAI) to the grain boundaries where the additives are attached, speeding up the crystal growth (Figure 1(c), III). Therefore, the energy barrier for the crystal coalescence can be significantly reduced and the perovskites are free to grow into larger grains with the assistance of urea and biuret. While in case of triuret, owing to its strong coordination ability with the perovskite precursors and its self-aggregation tendency (low solubility of triuret in the DMF solvent), it is prone to form enlarged clusters in the solution with reduced nucleus density, leading to subsequent exaggerated growth on a few nuclei. The imbalance of the rate for nucleation and crystal growth (nucleation<crystal growth) leads to the formation of dendritic morphologies [42]. Most of the precursors do not get the opportunity to interact with the additives and grow independently to reach a grain size similar to the pristine perovskite (Fig. S7).

### 2.3. Properties of Additives Assisted Perovskite Films

The elemental composition of the as-fabricated perovskite samples was characterized by an X-ray photoelectron spectroscopy (XPS). As shown in the core energy level spectrum of Pb *4f_7/2_* (Figure 2(i)), the peak position at 138.6 eV was related to the binding energy of lead in the perovskite. An additional peak at 138.0 eV was observed when using urea, biuret, and triuret as additives, which was ascribed to the formation of the Pb-O bond. This demonstrates a strong interaction between –C=O in additives and Pb in the perovskite, which is confirmed by Fourier transfer infrared spectroscopy characterization in Fig. S8. Regarding the core energy level spectrum of N *1*s as shown in Figure 2(j), the peak at 402.1 eV and 401.0 eV was assigned to the -NH_3_^+^ in perovskite and -NH_2_ in additives, respectively [43]. It was clearly observed that the intensity of the latter peak was enhanced following the order urea<biuret<triuret, with an atomic N/Pb ratio of 1.49, 1.74, and 4.45, respectively (Table S2). Therefore, the introduced additives of urea and biuret are mainly located at the grain boundaries of the perovskite crystallites, while triuret is concentrated at the surface, leading to a thin layer of triuret-associated dendritic perovskites.

A significant enhancement of PL emission intensity with an obvious red shift of the peak position and a prolonged charge carrier lifetime was observed upon the addition of urea and biuret additives (Figures 2(b) and 2(c)) due to the improved grain size and defect passivation by the additives. In contrast, the PL emission intensity and charge carrier lifetime for the film prepared using triuret as an additive were dramatically decreased and shortened, agreed with the photoluminescence microphotographs in Figures 2(e)–2(h)). It appears that the as-formed superficial dendritic perovskites can act as a luminescence quenching layer, which can be attributed to charge separation at the interface between bulk crystalline perovskite and dendritic perovskites. As confirmed by photoelectron yield spectroscopy (Fig. S9), the highest occupied molecular orbital (HOMO) levels of the perovskite films fabricated with urea and biuret additives showed negligible changes, while a clear deep shift by 0.2 eV was found when using triuret as an additive compared to the pristine perovskites. Given the consistent bandgap of these four types of perovskites as known from the same offset in the absorption spectrum, the lowest unoccupied molecular orbital (LUMO) energy level of dendritic perovskite was therefore lowered by 0.2 eV compared to the pristine perovskite. The effect is probably related to the oriented assembly of triuret at the surface of the perovskite with a concomitant offset of the band edges due to interfacial electric dipole formation.

As shown in Figure 2(d), the external photoluminescence quantum efficiency (*PLQE*) for all perovskite films is relatively low at low excitation densities but rises rapidly at high excitation densities. Low quantum yield indicates the existence of defect states through which nonradiative recombination is likely to happen. The increase of *PLQE* as a function of excitation fluence indicates a competition between nonradiative and radiative recombinations, and the latter one becomes dominant at high excitation density [44]. As aforementioned, the perovskite films deposited using urea and biuret as additives exhibited grain sizes (>1 *μ*m) much larger than the layer thickness (~400 nm) in which the photogenerated charges can cross the film through single grain other than boundaries, thereby reducing nonradiative recombination pathways and boosting the *PLQE* [45]. It has been reported that the –C=O and –NH_2_ groups in residual additives can interact with grain boundaries and passivate the defects by associating with negatively charged antisite defects (PbI_3_^−^) or halide ions and positively charged undercoordinated Pb^2+^ ions, leading to the *PLQE* enhancement. Owing to the above two benefits, significantly higher *PLQE* in biuret-doped perovskite is achieved for all excitation intensities, suggesting fewer nonradiative decay pathways.

### 2.4. The Effect of Additives on Device Performance

**Figure 3:**
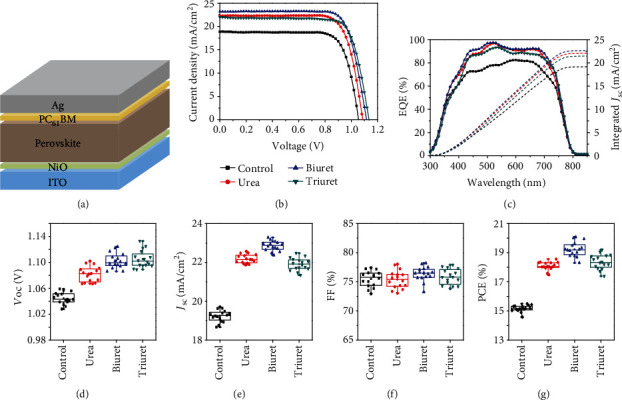
Device structure and photovoltaic performance. (a) Device configuration of the inverted perovskite solar cells (glass/ITO/NiO/MAPbI_3_/PC_61_BM/Ag). (b) Current density-voltage (*J*‐*V*) curves and (c) external quantum efficiency (EQE) spectrum of devices with different additives. (d–g) Statistical deviation of open-circuit voltage (*V*_OC_), short circuit current density (*J*_SC_), fill factor (FF), and PCE of the perovskite devices doped with different additives at LTTA condition (15 cells of each type).

Perovskite solar cells with an inverted device structure (Figure 3(a)) were prepared to investigate the effect of carbamide derivatives on photovoltaic performance. A typical PCE of 17% and 15% was obtained from the device based on pristine MAPbI_3_ perovskite when prepared using HTSA and LTTA, respectively, in par with published results of a device using similar configuration [37]. The efficiency difference was originating from different crystal quality when deposited using HTSA and LTTA (Fig. S6). Upon additive engineering, the device performance was significantly enhanced as clearly demonstrated in Figure 3 and Table S3 with reduced hysteresis and enhanced operational stability due to bonding capability between perovskite crystal lattice less bulk and interface defects in the device [14] (Fig. S10, S11). It is worth noting that even though the averaged performance was similar when the perovskites prepared using HTSA and LTTA through additive engineering, the reproducibility using LTTA was much higher than in the case of HTSA (Fig. S12) since the high-temperature solvent treatment is difficult to control. Hence, a simplified and low-cost deposition method was developed to prepare perovskite films towards efficient and cost-effective solar cells.

In particular, the functions of the incorporated additives on the device performance are quite different; for instance, the introduced urea and biuret can modify the crystallization dynamics for enlarged perovskite crystal growth with minimized grain boundaries; the incorporated triuret is prone to aggregation leading to the formation of dendritic perovskites at the perovskite/PC_61_BM interface, which can act as energy cascade layer between the perovskite and PC_61_BM, facilitating the electron transport from perovskite to electron transport layer; the –C=O and –NH_2_ in residual additives can further passivate intrinsic and surface defects to reduce nonradiative recombination channels. The above benefits from additive engineering accounted for the significant improvement of *V*_OC_ and *J*_SC_ (Figure 3(b)). The convolution of the EQE (Figure 3(c)) with the standard AM1.5G solar spectrum agreed well with the *J*_SC_ values taken from *J*‐*V* measurements. Moreover, it was noted that the EQE improvement was originated from the whole wavelength region due to the reduced surficial charge recombination and facilitated charge transfer, and the *V*_OC_ increase was attributed to the enhanced crystal quality and reduced energy barrier for the charge transport. After probably device optimization (Table S4, Fig. S13), the biuret-doped perovskites present the best performance with a champion efficiency of 20.1%, compared to 19.2%, 18.6% of the devices based on triuret and urea, respectively. The reproducibility of the device based on different additives was evaluated by collecting the performance of 15 cells from four different batches, and the statistical deviation of each parameter was summarized in Figures 3(d)–3(f), which indicated good reproducibility.

### 2.5. Synergistic Benefits from Mixed Additives

**Figure 4:**
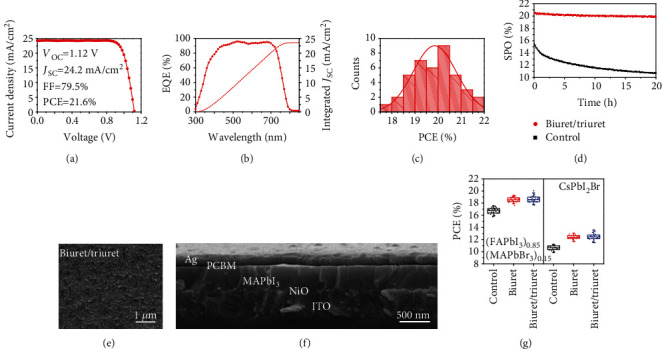
Performance of the perovskite device with mixed additives. (a) *J*‐*V* curve. (b) EQE spectrum of the champion device. (c) PCE histogram of the device prepared by adding mixed biuret/triuret as an additive. (d) The stabilized power output (SPO) of the champion device and the control device. (e, f) Top SEM and cross-sectional SEM of the perovskite film prepared with mixed biuret/triuret as an additive. (g) Effect of additives on the photovoltaic performance of mixed (FAPbI_3_)_0.85_(MAPbBr_3_)_0.15_ perovskite and inorganic CsPbI_2_Br perovskite with a structure of ITO/PTAA/(FAPbI_3_)_0.85_(MAPbBr_3_)_0.15_/PC_61_BM/Ag and ITO/NiO/CsPbI_2_Br/PC_61_BM/Ag, respectively.

In order to bring synergistic benefits from additive engineering, we tried to mix additives with different combinations in terms of urea/triuret, biuret/triuret, or urea/biuret/triuret into the precursor to further enhance the device performance. The performance metrics of the above devices are summarized in Table S5. The best performing device was achieved by using the additive mixtures of biuret/triuret, and a champion cell with PCE of 21.6%, *J*_SC_ of 24.2 mA/cm^2^, *V*_OC_ of 1.12 V, and FF of 79.5% was achieved as detailed in Figure 4(a). To the best of our knowledge, this is the highest PCE value of solar cells based on pure MAPbI_3_ perovskite in an inverted device structure. As shown in the EQE spectra of Figure 4(b), the improvement of *J*_SC_ is ascribed to the EQE increase near the bandgap region from 550 nm to 750 nm compared to the device with an additive of biuret (Figure 3(c)) owing to the reduced surficial charge recombination and facilitated charge transfer. It was noted that the perovskites prepared with mixed additives present a dendritic surface morphology (Figure 4(e)) and enlarged grain size as seen from the cross-sectional SEM in Figure 4(f), which was similar to that of triuret and biuret additives, respectively. Therefore, the benefits of enhanced crystal quality and facilitated charge transfer from an additive of biuret and triuet can be combined by rationally mixing the additives.

As shown in Figure 4(e), the device stability was also dramatically enhanced by using mixed additives, and only a slight decay (less than 5%) was found during 20 hours of operation, compared to 30% decay of the control device. And also, the device based on biuret/triuret additives showed the best long-term storage stability in N_2_ and ambient environment due to the enhanced crystal quality and interface properties against the oxygen and moisture penetration (Fig. S14).

### 2.6. Method Exploration in Other Types of Perovskites

To check if the additive engineering by carbamide derivatives is a general strategy that can be applied to improve the crystal quality of other types of perovskites, the additives of biuret were preliminarily tried in the mixed perovskite (FAPbI_3_)_0.85_(MAPbBr_3_)_0.15_ and the inorganic perovskite CsPbI_2_Br. As shown in Fig. S15, the grain size of both (FAPbI_3_)_0.85_(MAPbI_3_)_0.15_ and CsPbI_2_Br perovskites was significantly enlarged by using biuret as additives. As demonstrated in Figure 4(g) and supplementary Fig. S16, the performance of the device in an inverted structure was also significantly enhanced by adding biuret and biuret/triuret as additives, respectively. A similar trend was found that the device based on mixed biuret/triuret additives was performing better than the one using biuret due to the facilitated electron transfer at perovskite/PC_61_BM interface. In contrast, a regular device architecture of ITO/TiO_2_/(FAPbI_3_)_0.85_(MAPbI_3_)_0.15_/Spiro-OMeTAD/Au was also tested (Fig. S17). The device performance was boosted by employing biuret as an additive owing to the improved quality of the perovskite layer. However, when using the combined biuret/triuret molecules as additives, the device performance declined owing to a reduced fill factor, which is ascribed to an unfavorable interfacial energy level alignment at perovskite/dendritic perovskite/spiro-OMeTAD interface. The as-formed dendritic perovskite layer is beneficial for the electron transfer from the perovskite to the electron transport layer, while the hole transfer from the perovskite to spiro-OMeTAD is unfavored.

## 3. Conclusions

In conclusion, the incorporation of a small amount of carbamide derivatives, such as urea, biuret, and triuret, as additives in perovskite precursor is an efficient way to simplify the fabrication process and boost device performance. This strategy can be implemented into different types of perovskites such as MAPbI_3_, mixed perovskite, or inorganic perovskite. The mechanism of action, however, is specific to the additive. For instance, urea and biuret are beneficial for the large grain crystal growth due to the association between the –C=O and –NH_2_ groups with the PbI_2_ and MAI. Residual urea and biuret can further passivate the intrinsic defects and eliminate nonradiative charge recombination. In contrast, the incorporated triuret molecules are prone to aggregation and assemble at the perovskite surface to form a cascade junction, thereby reducing the energy barrier for the electron transfer from the perovskite to the electron transport layer and increasing the PCE of the devices. By rationally combining the additives of biuret and triuret, the as-deposited perovskite solar cells can bring synergistic benefit from the additives, resulting in the highest efficiency of 21.6% in an inverted device structure. The simplified fabrication procedure will provide a simple way to fabricate high-performance perovskite solar cells.

## 4. Materials and Methods

### 4.1. Materials

The perovskite precursors in terms of lead iodide (PbI_2_), lead bromide (PbBr_2_), methylammonium iodide (MAI), formamidinium iodide (FAI), methylammonium bromide (MABr), and cesium iodide (CsI) were purchased from Xi'an Polymer Light Technology Corporation and used as received. The carbamide molecules (including urea, biuret, and triuret) were purchased from Sigma-Aldrich and purified by recrystallization.

### 4.2. Solar Cell Fabrication

The glass/ITO substrates were washed by sonicating in a cleaning agent, deionized water, acetone, and isopropanol solvent for 10 min, respectively. The NiO layer was prepared by spin coating the mixed solution of nickel (II) nitrate hexahydrate solution in ethylene glycol (1 M) with ethylenediamine (1 M) with the speed of 2500 r.p.m for 90 s in ambient air and followed by a high-temperature thermal annealing at 300°C for 1 h. Then, the NiO-coated substrates were moved into a glove box filled with nitrogen to complete all the other fabrication steps. The perovskite precursors were prepared by mixing 1.25 mmol PbI_2_ and MAI in 1 mL mixed solvent of DMF and DMSO with a volume ratio of 4 : 1. For the one-additive system, each additive (0.05 mmol) was then added into 1 mL precursor solution. And for the mixed-additive system, 0.05 mmol biuret and 0.01 mmol triuret were added into a 1 mL perovskite precursor. To deposit the perovskite layer, the precursor solution was dropped onto the as prepared glass/ITO/NiO substrates, and 300 *μ*L chlorobenzene with 6 mg/mL PC_61_BM was used as an antisolvent and quickly dropped on the sample surface at around 8^th^ second. For the LTTA method, the films were annealed at 60°C for 5 minutes, and for the HTSA method, the films were annealed at 100°C for 10 minutes in the DMF atmosphere to form the MAPbI_3_ layers. After that, ~40 nm thick PC_61_BM was spin-coated on the MAPbI_3_ layer as an electron transport layer. In the end, 120 nm thick silver was thermally deposited under a high vacuum condition (<5.0 × 10^−4^ Pa) as electrodes. For (FAPbI_3_)_0.85_(MAPbBr_3_)_0.15_ precursors, firstly, 1.49 mmol PbI_2_ and FAI, 0.26 mmol PbBr_2_ and MABr solutions were dissolved in 1 mL mixed solvent consisting of DMF and DMSO (4 : 1 in volume) and stirred at 70°C for an hour to form the perovskite precursor. For the one-additive system, 0.05 mmol biuret was then added into a 1 mL perovskite precursor and stirred at 70°C for 30 minutes. And for the mixed-additive system, 0.05 mmol biuret and 0.01 mmol triuret were added into 1 mL perovskite precursor solution and stirred at 70°C for an hour before usage. The mixed perovskites were prepared with a spin-coating speed of 6000 r.p.m. for 30 s, and 50 *μ*L of diethyl ether was used as an antisolvent at 25^th^ second and followed by a thermal annealing at 100°C for an hour to form the mixed (FAPbI_3_)_0.85_(MAPbBr_3_)_0.15_ perovskite layer.

For CsPbI_2_Br, perovskite precursors were prepared by dissolving 184 mg PbI_2_, 146 mg PbBr_2_, and 208 mg CsI in 1 mL DMF. For the one-additive system, 0.05 mmol biuret was then added into 1 mL perovskite precursor and stirred at 60°C for 30 minutes. And for the mixed-additive system, 0.05 mmol biuret and 0.01 mmol triuret were added into a 1 mL perovskite precursor and stirred at 60°C for an hour. The perovskite film was depositing with a spin speed of 2500 r.p.m. for 30 s and annealed at 120°C in N_2_ atmosphere for 10 minutes to form the CsPbI_2_br layer.

### 4.3. Thin-Film Characterization

The XRD pattern was analyzed by a thin film X-ray diffractometer (SmartLab) with CuK*α* radiation. The FTIR spectra were characterized using Fourier transform infrared spectroscopy (Thermo Fisher Scientific, IS50) with a reflection mode. Top-surface SEM images and cross-sectional SEM images were taken by a scanning electron microscope (JEOL, JSM-7800F), and the photoluminescence microphotographs were measured with an excitation light source of 530 nm. Photoluminescence (PL) spectra and time-resolved photoluminescence (TRPL) spectra were measured by a photoluminescence spectrometer (Edinburgh Instruments, FLS 980) excited with a 531 nm laser. The HOMO level measurement of the perovskite films was carried out by using an Ionization Energy Measurement System (Model IPS-4, Nanjing SunnyTech Ltd., China). The X-ray photoelectron spectrum (XPS) measurements were conducted by an X-ray photoelectron spectroscopy (UIVAC-PHI, PHI 5000 VersaProbe).

### 4.4. Device Characterization

The photovoltaic measurements in terms of *J*‐*V* curves and steady-state performance of the solar cells were carried out in the glove box at a standard one sunlight illumination condition (AM 1.5 G, with a light intensity of 100 mW·cm^−2^) that combines a Keithley 2400 source meter with a solar simulator (Enlitech, SS-F5-3A), calibrated with a standard silicon solar cell (Enlitech, SRC-2020). The *J*‐*V* curve of the solar cells was measured in a reverse scanning mode with a step difference of 0.02 V. External quantum efficiency (EQE) spectra were characterized with a homemade instrument that combines an integrating sphere (Ocean Optics) and spectrograph (QE6PRO, Ocean Optics).

## Supplementary Materials

Supplementary Materials Additive selection, photovoltaic performance, ^1^H NMR spectra, color changes, XRD spectra, top-surface SEM and cross-sectional SEM, FTIR spectra, IPS, device performance when prepared at LTTA and HTSA, hysteresis, steady-state power-out, reproducibility, shelf-storage stability of the perovskite devices doped with different carbamide molecules, and device performance of mixed perovskite and inorganic CsPbI_2_Br perovskites with additives (Figures S1-S17, Tables S1-S5) are included.
