# Supplementary material for: All-in-One Deposition to Synergistically Manipulate Perovskite Growth for High-Performance Solar Cell
Source: Research (Wash D C). 2020 Oct 14;2020:2763409. doi: 10.34133/2020/2763409 (PMC7582804; doi:10.34133/2020/2763409)
Supplement: Supplementary Materials — Additive selection, photovoltaic performance, 1H NMR spectra, color changes, XRD spectra, top-surface SEM and cross-sectional SEM, FTIR spectra, IPS, device performance when prepared at LTTA and HTSA, hysteresis, steady-state power-out, reproducibility, shelf-storage stability of the perovskite devices doped with different carbamide molecules, and device performance of mixed perovskite and inorganic CsPbI2Br perovskites with additives (Figures S1-S17, Tables S1-S5) are included. [file 2763409.f1.docx]

Supporting Information

**All-in-one deposition to synergistically manipulate perovskite growth for high performance solar cell**

Yifan Lv, Hui Zhang*, Jinpei Wang, Libao Chen, Lifang Bian, Zhongfu An*, Zongyao Qian, Guoqi Ren, Jie Wu, Frank Nüesch, Wei Huang*

**Supplementary S1| Additives selection**

Besides urea, biuret and triuret, a series of small molecules similar with biuret were tried as additives in the perovskite preparation, and their chemical structures were shown in Fig. S1. Compared to biuret, the doping of additive **2** and **3** present an opposite direction in the perovskite growth, the perovskite crystallization was slowed down as shown in Fig.S4. It seems that the -C=O group plays an important role in affecting the nucleation and growth dynamics and promote perovskite crystal growth, and the existence of the -NH_3_ group in the molecular retarded the crystallization.

A series of perovskite solar cells with an inverted device architecture (Fig.3a) of ITO/NiO/MAPbI_3_/PC_61_BM/Ag were fabricated, where the perovskite layers were prepared using different additives. As summarized in Tab. S1, the molecule **2** and **3** showed negative effects on device performance due to the retarded crystallization process. Among the additives of molecule **4**, **5**, **6** and biuret, the best performance was achieved when using biuret as additives, indicating the importance of the coexistence of both end -NH_2_ and intermediate -NH- groups.


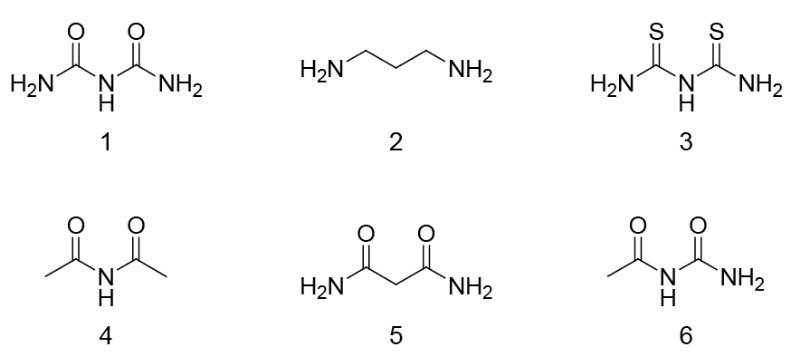


**Figure S1.** molecular structure of the urea derivatives used as additives

**Table S1.** photovoltaic performance of the device based on perovskite with different additives under structure of ITO/NiO/MAPbI_3_/PC_61_BM/Ag

| **Sample** | **V_OC_**  **(V)** | **J_SC_**  **(mA/cm^2^)** | **FF**  **(%)** | **PCE**  **(%)** |
| --- | --- | --- | --- | --- |
| Control | 1.06 | 19.5 | 75.0 | 15.5 |
| 1 | 1.09 | 23.1 | 76.5 | 19.2 |
| 2 | 0.915 | 3.12 | 29.5 | 0.84 |
| 3 | 0.995 | 9.59 | 23.2 | 2.22 |
| 4 | 1.03 | 21.3 | 69.6 | 15.2 |
| 5 | 1.08 | 23.0 | 71.6 | 17.8 |
| 6 | 1.09 | 23.5 | 70.4 | 18.0 |


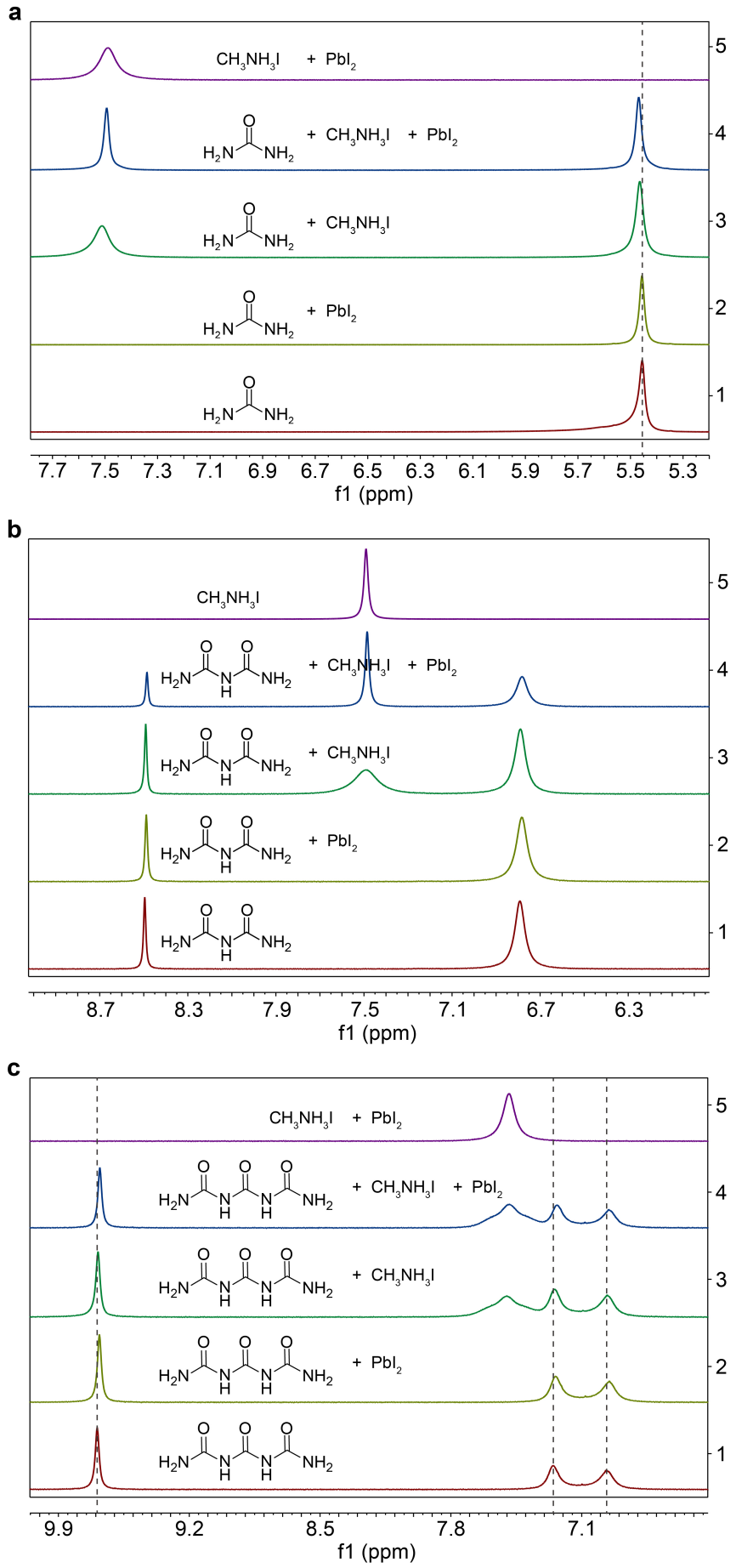


**Figure S2.** ^1^H NMR spectra of MAI, MAI+PbI_2_, MAI+PbI_2_+additives, MAI+additives, PbI_2_+additives and additives, where the additive is (a) urea, (b) biuret, (c) triuret.


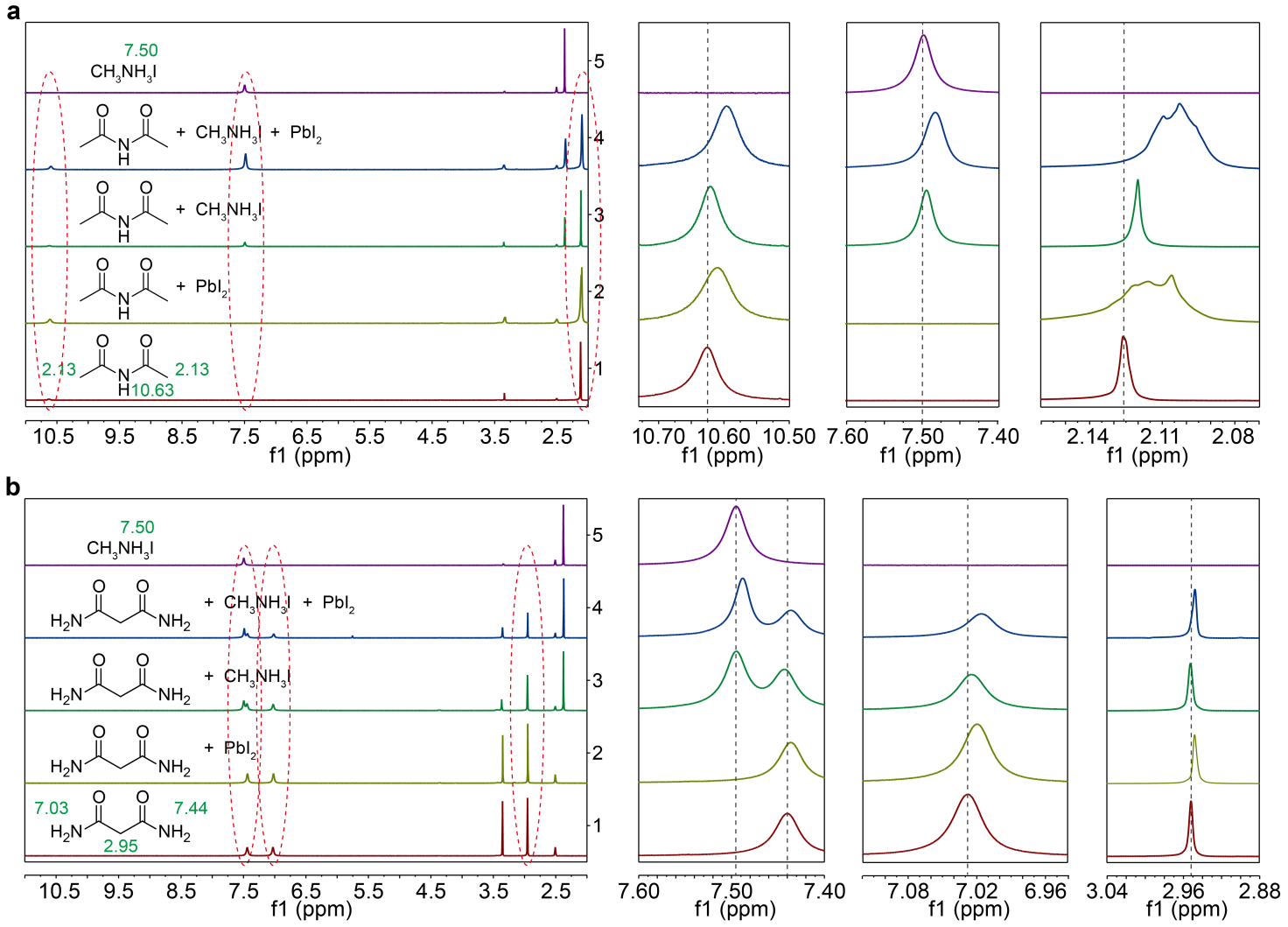


**Figure S3.** ^1^HNMR spectra of MAI, MAI+PbI_2_, MAI+PbI_2_+additives, MAI+additives, PbI_2_+additives and additives, where the additive is (a) molecule 4 and (b) molecule 5.


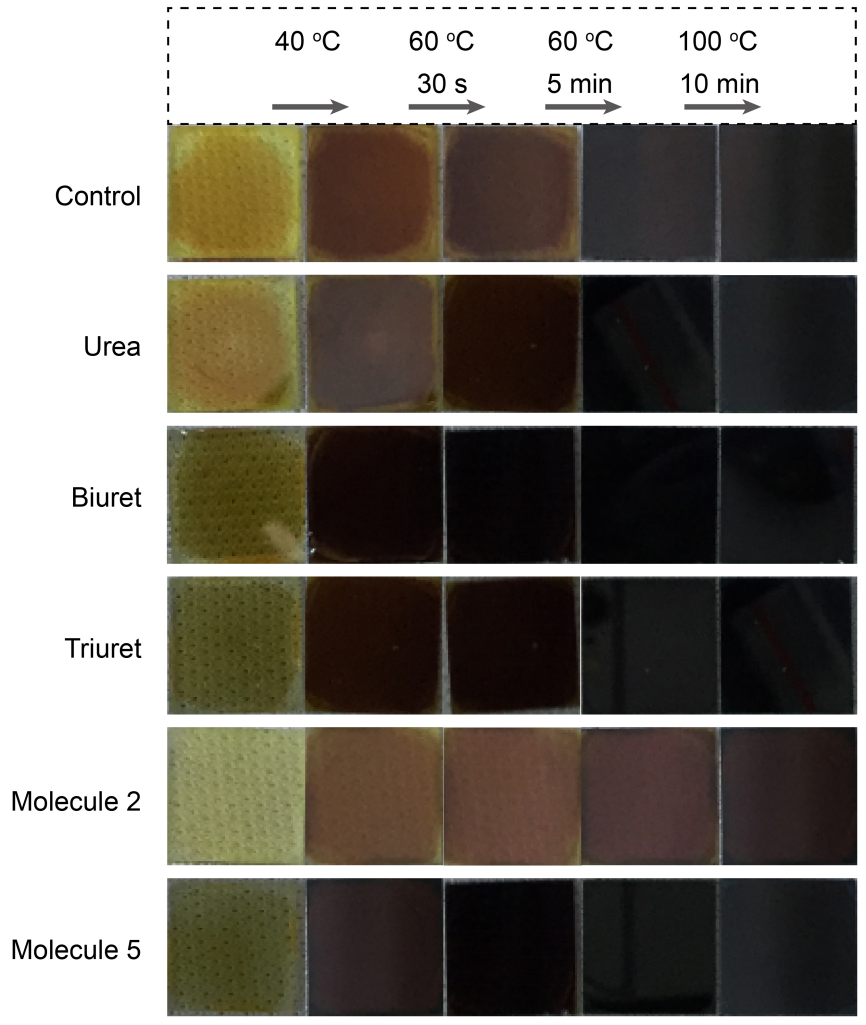


**Figure S4.** Color changes of the perovskite films prepared using different additives during the post-annealing process.


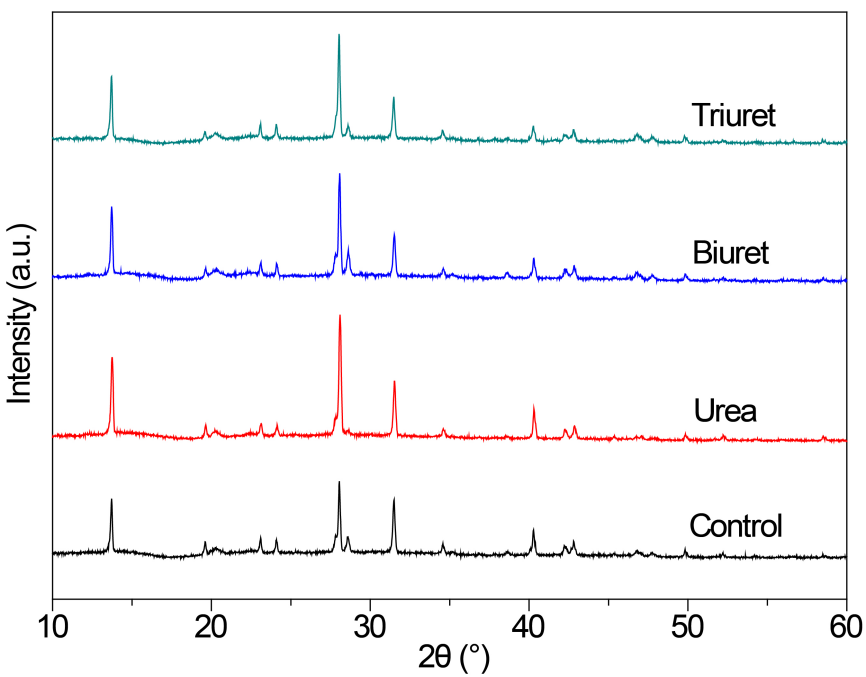


**Figure S5.** X-ray diffraction (XRD) spectra of MAPbI_3_ films prepared with different additives at high temperature solvent annealing.


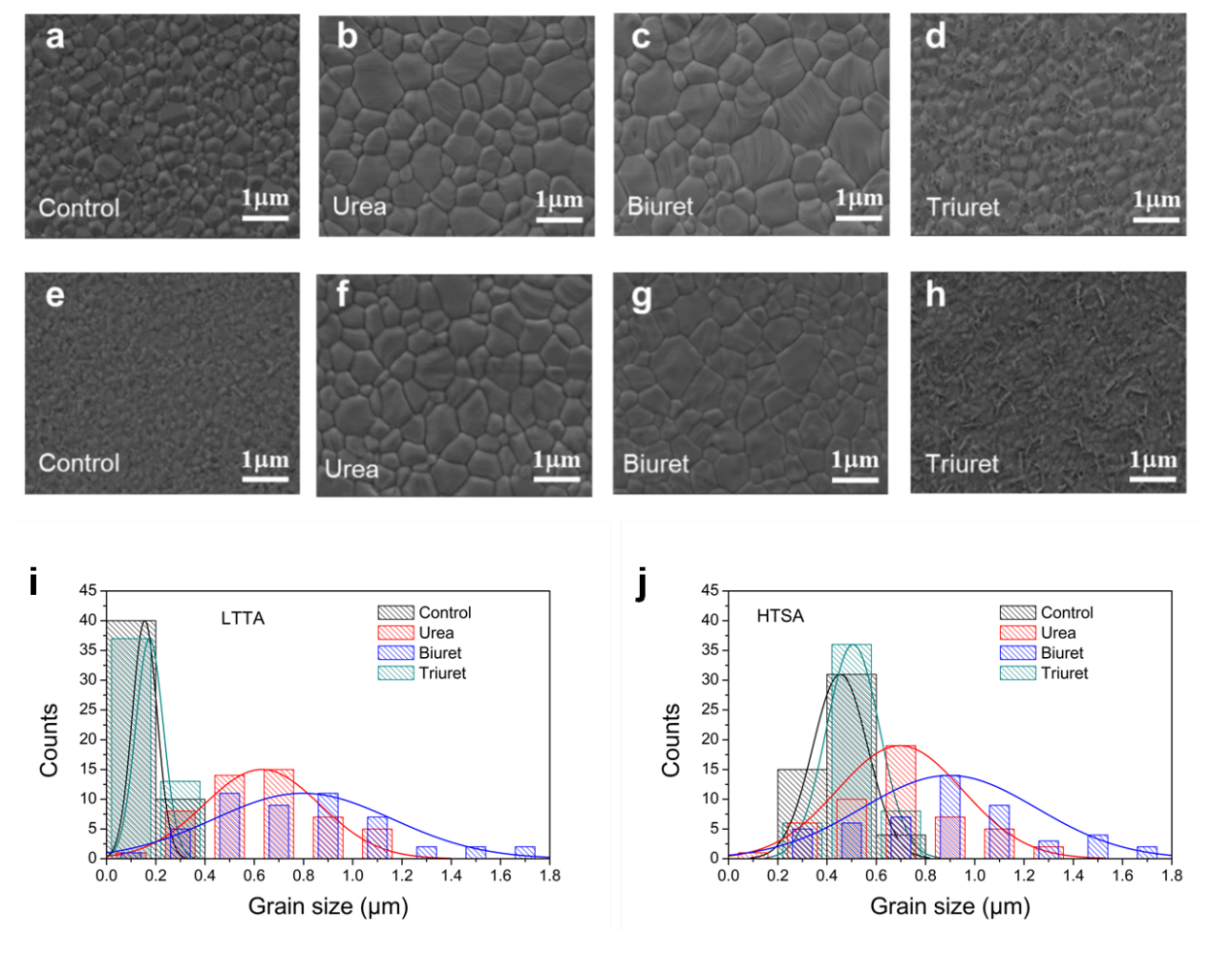


**Figure S6.** Scanning electron microscopy (SEM) images of perovskite films prepared without additives or with urea, biuret and triuret as additives, the first row (a, b, c, d) was prepared at HTSA and the second row (e, f, g, h) was that of at LTTA, and the histogram of grain sizes at (i) LTTA and (j) HTSA.


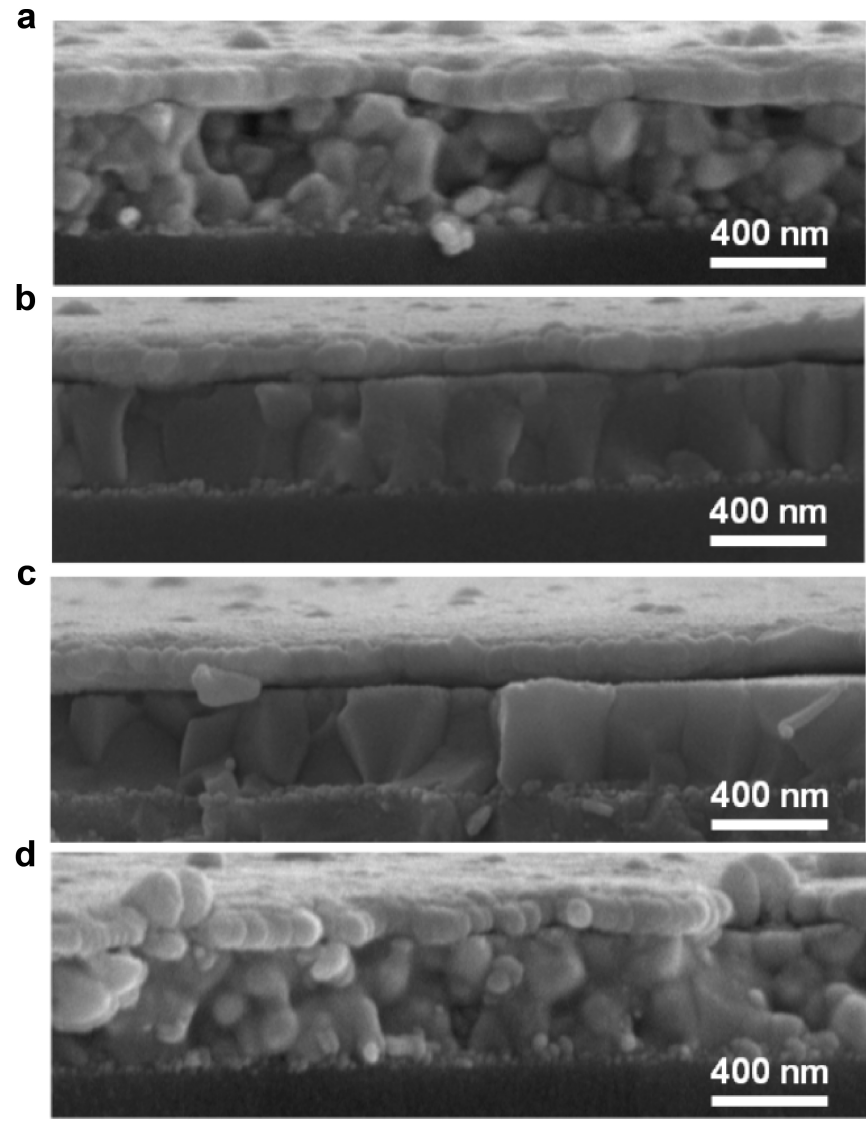


**Figure S7.** Cross sectional scanning electron microscopy (SEM) images of perovskite films prepared (a) without additives and with (b) urea and (c) biuret and (d) triuret as additives at LTTA.

**Table S2.** N and Pb content at the surface of MAPbI_3_ films prepared with different additives at LTTA, measured by XPS spectra.

| **Additive** | **N(%)** | **Pb(%)** | **N/Pb** |
| --- | --- | --- | --- |
| Control  Urea  Biuret  Triuret | 9.76  9.58  12.44  17.19 | 8.58  6.43  7.15  3.86 | 1.14  1.49  1.74  4.45 |

In ideal case, the mole ratio of N/Pb value should be equal to 1 in perovskite crystal, and the high N/Pb value in the sample meant that more additives remained at the surface. As summarized in the above table, the mole ratio of N/Pb is higher than that of the control sample and increased in the order of urea<biuret<triuret.


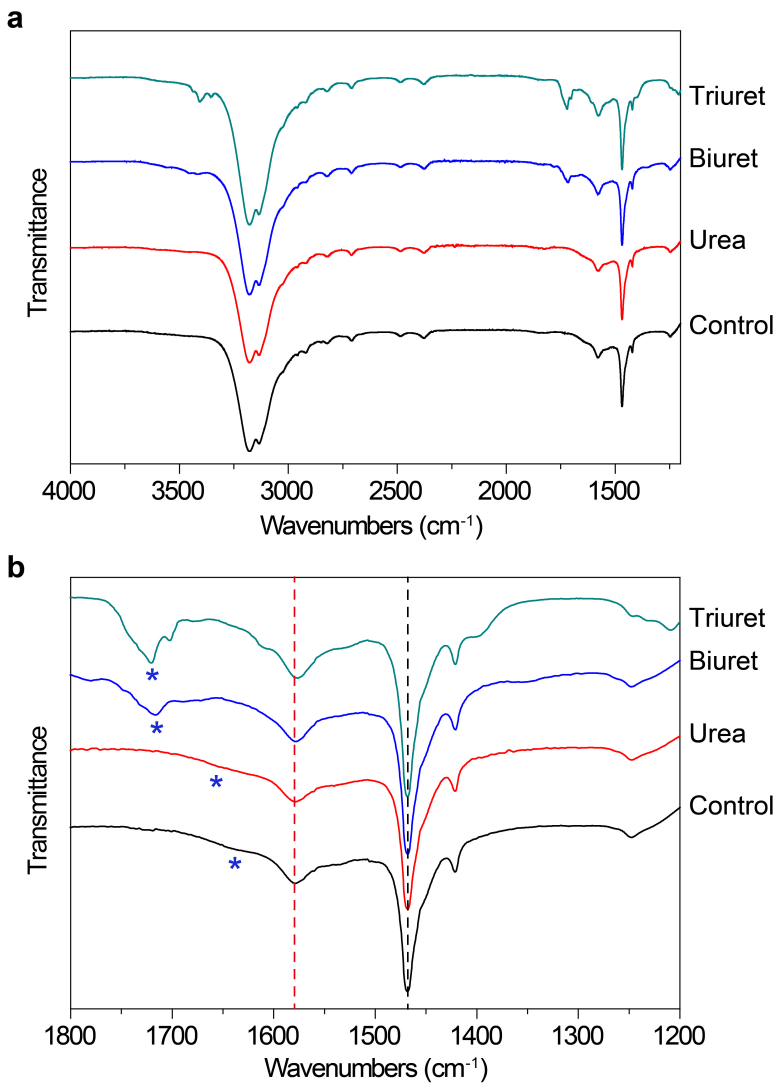


**Figure S8.** Fourier transform infrared (FTIR) spectra of MAPbI_3_ films prepared with different additives at LTTA, ranged from (a) 4000-1000cm^-1^ and (b) 1800-1200cm^-1^. Where, C=O stretch (blue asterisk), N–H bend (red dotted line), and C–N stretch (black dotted line). The typical signal of N–H bend, and C–N stretch of pristine MAPbI_3_ and perovskite fabricated with additives were located at the same wavenumbers. As observed similarly with reported results [1], a slight blue shift on C=O stretch in the sample with Urea due to the interaction between the residual -C=O in Urea and perovskite crystal elements. More extent of blue-shift on C=O stretch appears in the samples with biuret and triuret, suggesting stronger interaction between the residual additives with perovskite crystals.


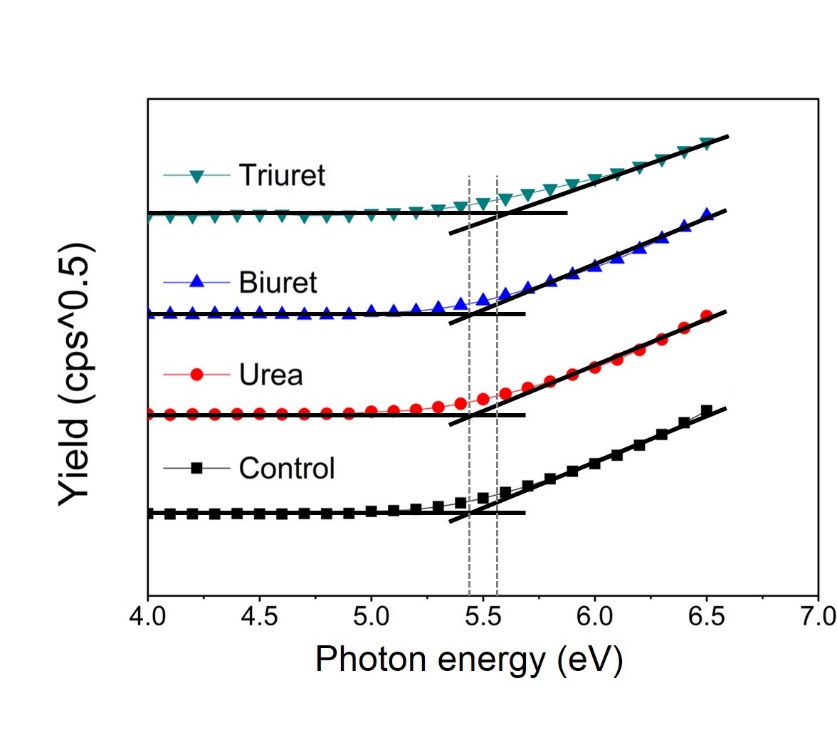


**Figure S9.** Photoelectron yield spectroscopy characterizations of MAPbI_3_ films prepared with different additives at LTTA.

**Table S3.** Performance comparison of perovskite solar cells when prepared at LTTA and HTSA. Averaged from 16 individual cells.


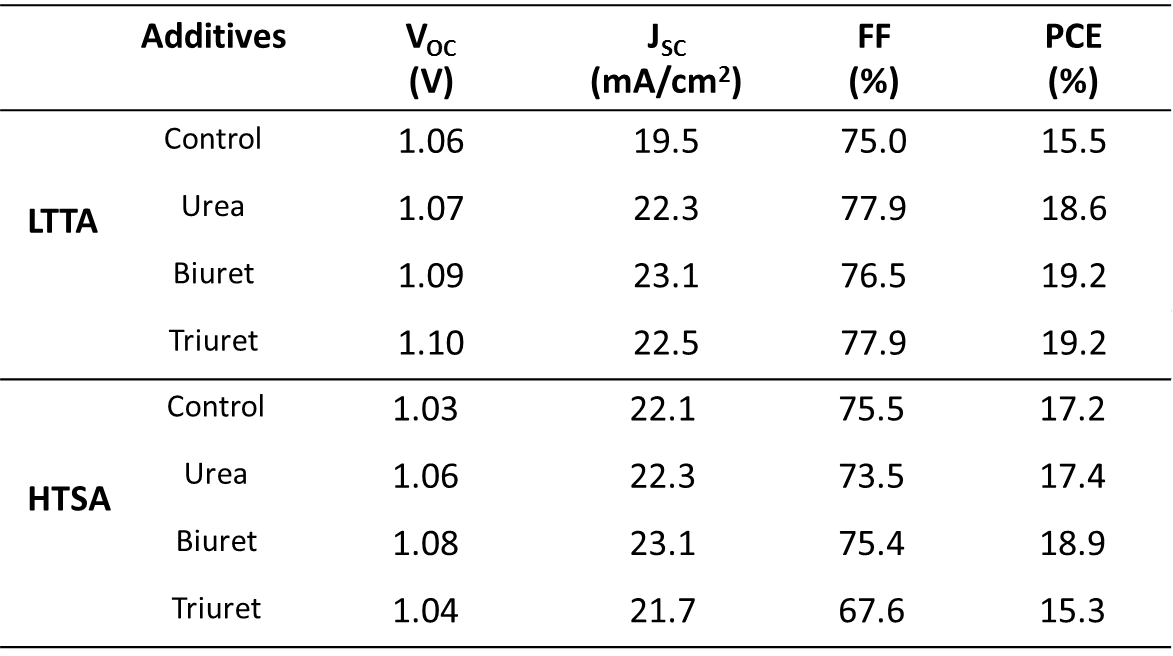


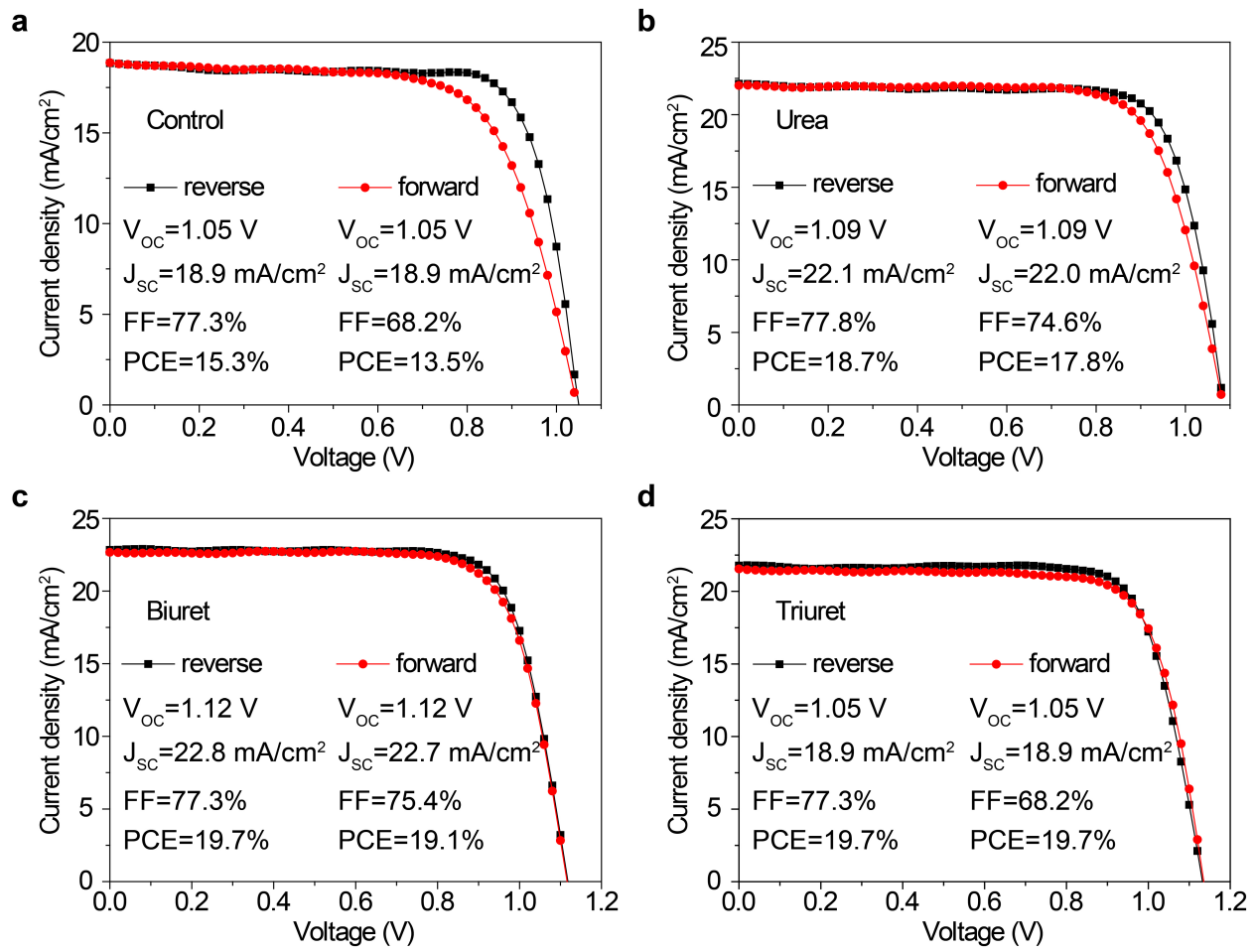


**Figure S10.** *J–V* curves of the perovskite solar cells prepared with (a) no additive (b) Urea (c) Biuret and (d) Triuret at LTTA, measured in both reverse and forward scanning directions.


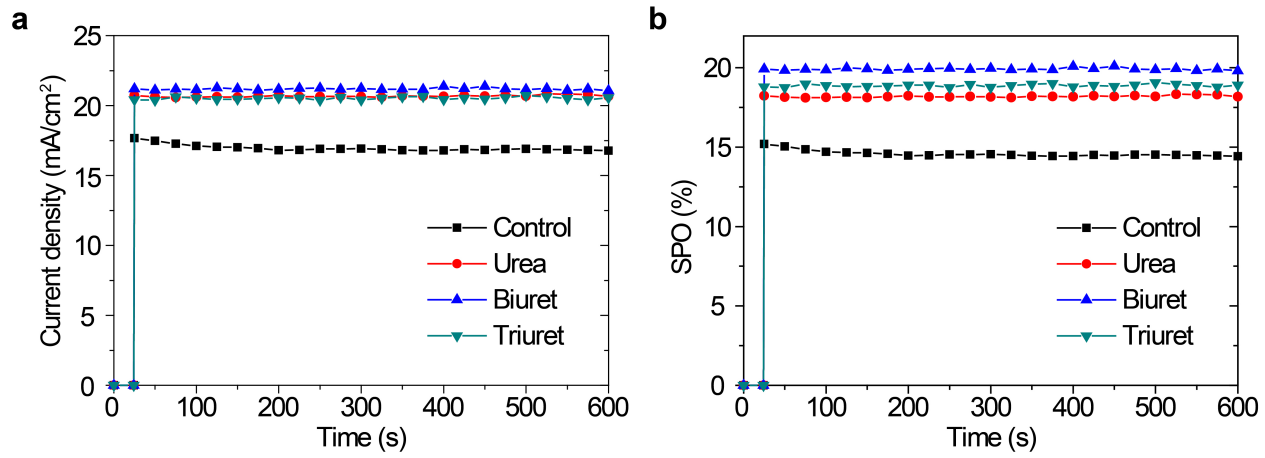


**Figure S11.** Steady-state (a) photocurrent and (b) efficiency of the perovskite solar cells prepared with different additives at LTTA.


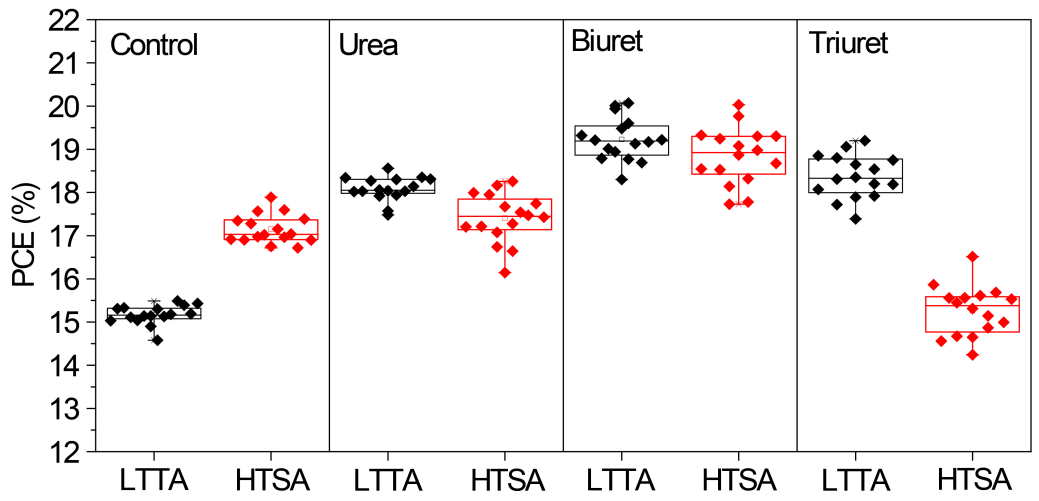


**Figure S12.** Plot of PCE deviation of the perovskite device prepared with different additives at HTSA and LTTA, respectively. X mark means the maximum value and minimum value, small check inside marks the mean value.

**Table S4.** Photovoltaic performance of the devices with different content of Biuret, and all perovskites were prepared by LTTA for 5 min. Each group was averaged from 16 devices.

| **Biuret content (mg/ml)** | ***V_oc_* (V)** | ***J_SC_* (mA/cm^2^)** | **FF (%)** | **PCE (%)** |
| --- | --- | --- | --- | --- |
| 0  2.5 (0.025M)  5 (0.05M)  7.5 (0.075M)  10 (0.1M) | 1.06  1.08  1.11  1.09  1.01 | 19.2  22.1  22.7  23.1  22.4 | 75.4  75.8  76.4  74.9  69.8 | 15.3  18.1  19.3  18.9  15.8 |

The molar ratio of introduced additives to the perovskite precursor is another determining factor of crystallization dynamics, which was varied to optimize the device performance. The photovoltaic parameters of the devices based on perovskites with different amounts of biuret are summarized in Table S4. An optimum molar ratio of biuret to perovskite precursor was found to be around 1:25, and a higher doping concentration was detrimental to the device performance due to the overgrowth of the crystals resulting in fractures and pinholes in the films (Figure S13).


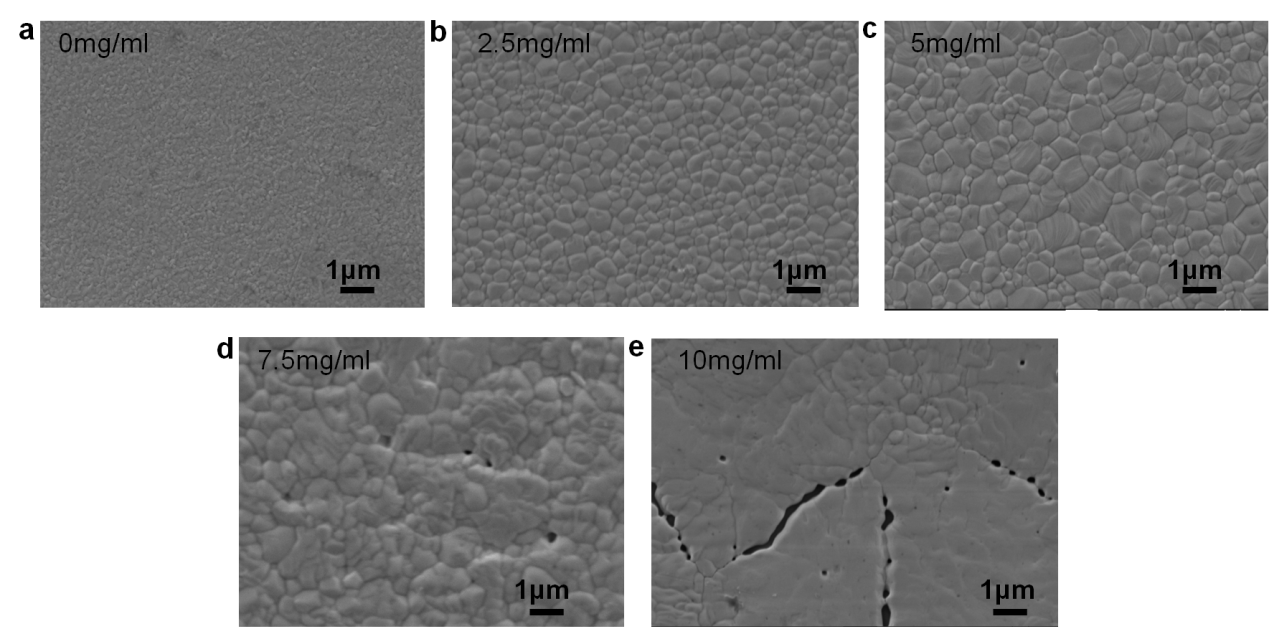


**Figure S13.** Top-surface SEM images of MAPbI_3_ films prepared by precursor with different content of Biuret at LTTA: (a) 0mg/ml, (b) 2.5mg/ml, (c) 5mg/ml, (d) 7.5mg/ml and (e) 10mg/ml. To observe the actual situation in the devices, all perovskite films were prepared on NiO layer for SEM measurements. It can be observed that the grain size is increasing with the increasing of added biuret content. As the Biuret content was increased to 7.5mg/ml (0.075M) or more, some huge grains with pin-holes and gullies appeared. The pin-holes and gullies were detrimental to the device performance by introducing grain boundary recombination loss.

**Table S5.** Device performance comparison of perovskite prepared with different additive combinations

| **Additive combinations** | **V_OC_**  **(V)** | **J_SC_**  **(mA/cm^2^)** | **FF**  **(%)** | **PCE**  **(%)** |
| --- | --- | --- | --- | --- |
| urea | 1.082 | 22.18 | 75.41 | 18.09 (18.56) |
| urea/triuret | 1.084 | 23.02 | 75.66 | 18.88 (19.50) |
| biuret | 1.102 | 22.85 | 76.34 | 19.23 (20.07) |
| biuret/triuret | 1.105 | 23.35 | 76.17 | 19.65 (20.99) |


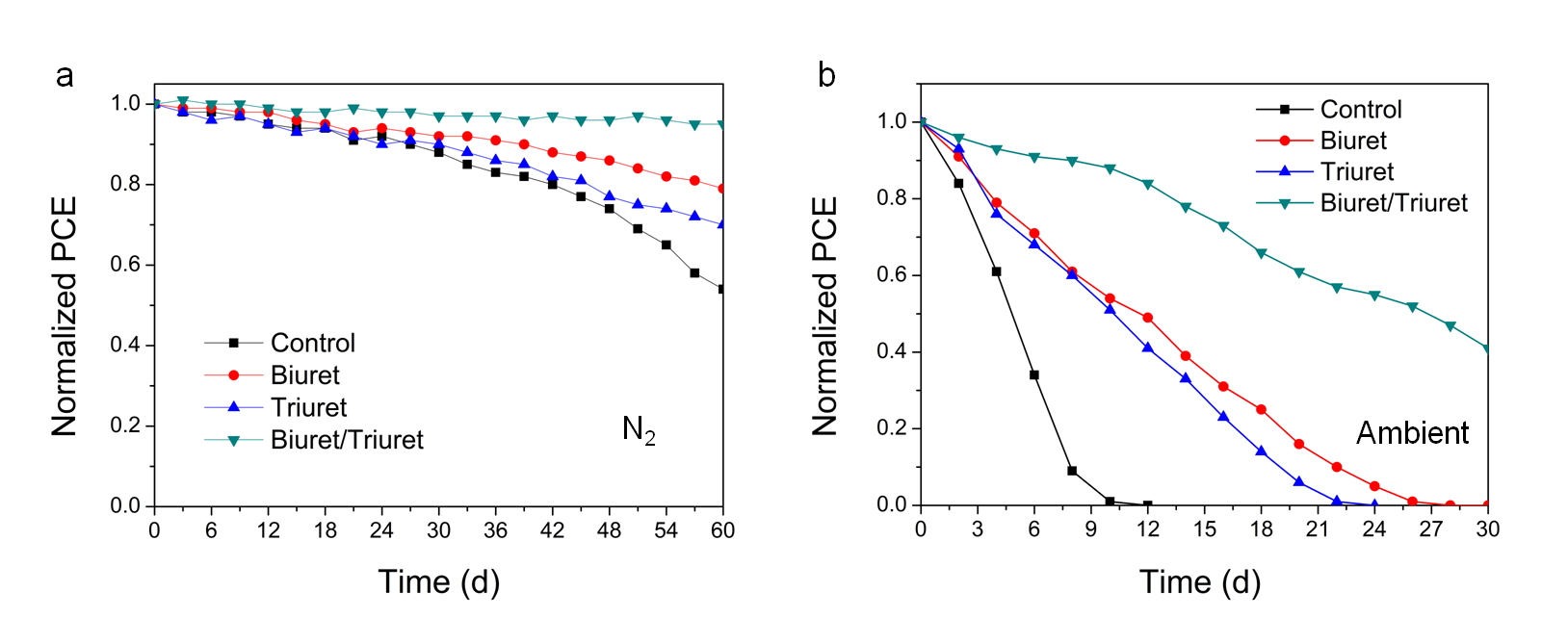


**Figure S14.** Storage stability of the devices based on perovskites with different additives when stored at (a) N_2_ environment and (b) ambient environment.


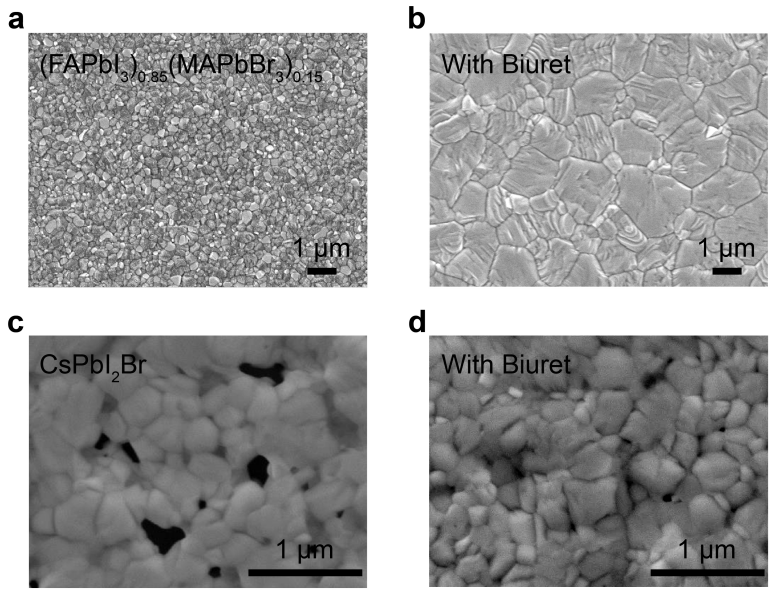


**Figure S15.** SEM images of (a, b) (FAPbI_3_)_0.85_(MAPbBr_3_)_0.15_ perovskites and (c, d) inorganic CsPbI_2_Br perovskites prepared (a, c) without or (b, d) with biuret as additive.


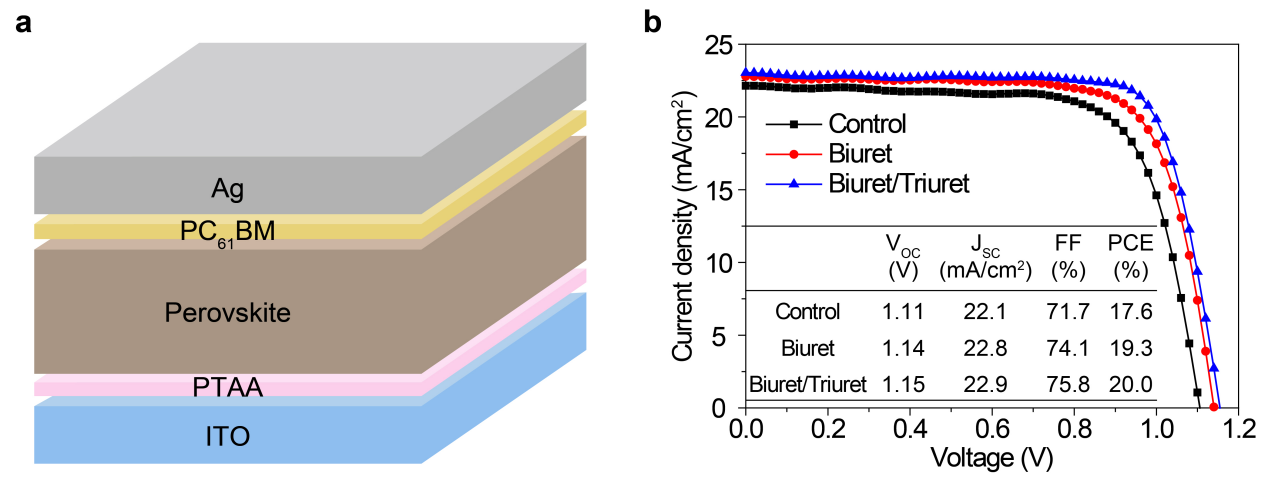


**Figure S16.** *J–V* curves of the devices with structure of ITO/PTAA/(FAPbI_3_)_0.85_(MAPbBr_3_)_0.15_/PC_61_BM/Ag, where the perovskites were prepared without or with biuret and biuret/triuret as additive.


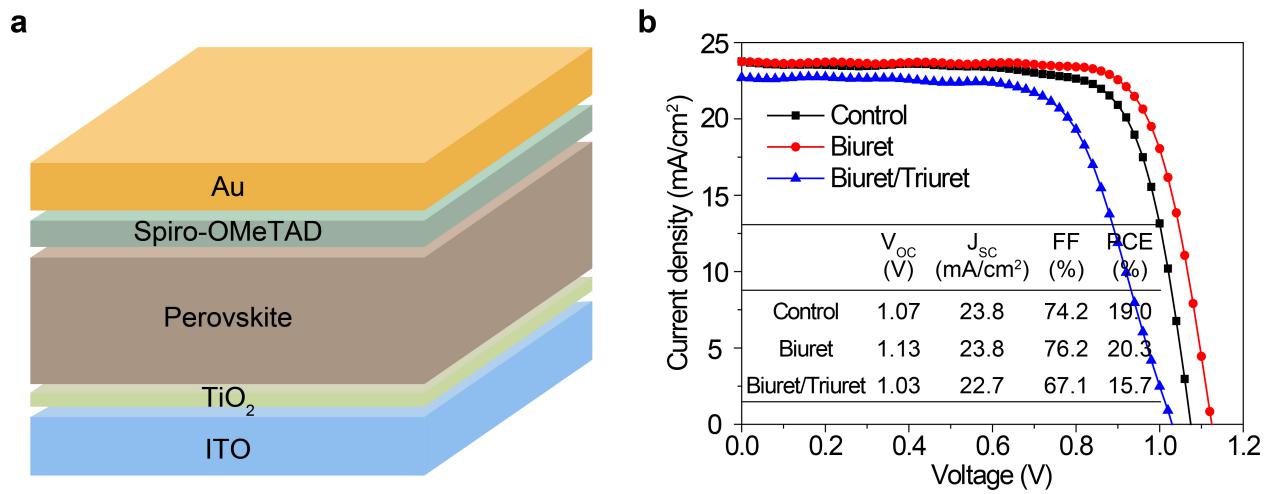


**Figure S17.** *J–V* curves of the devices with structure of ITO/TiO_2_/(FAPbI_3_)_0.85_(MAPbBr_3_)_0.15_/Spiro-OMeTAD/Au, where the perovskites were prepared without or with biuret and biuret/triuret as additive.

**References:**

[1] J. D. Lee, S. H. Bae, Y. T. Hsieh, et al, “A Bifunctional Lewis Base Additive for Microscopic Homogeneity in Perovskite Solar Cells,” *Chem*, vol. 3, no. 2, pp. 290-302, 2017.
